# Supplementary figures and images for: Seasonal temperature variation influences climate suitability for dengue, chikungunya, and Zika transmission
Source: PLoS Negl Trop Dis. 2018 May 10;12(5):e0006451. doi: 10.1371/journal.pntd.0006451 (PMC5963813; doi:10.1371/journal.pntd.0006451)

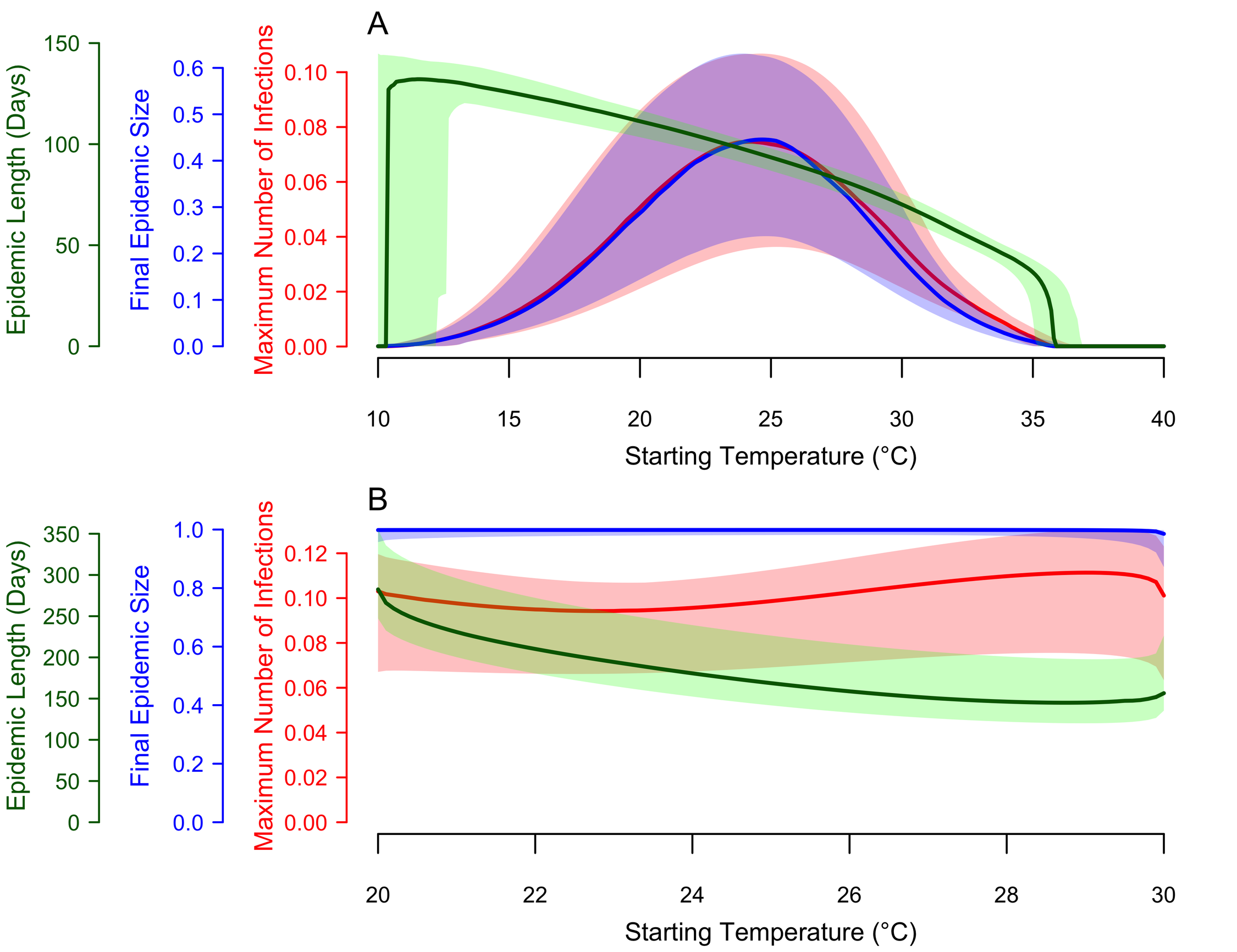

Supplement: S1 Fig — The red curve represents the median maximum number of humans in the infected class (IH) at any given point during the simulation. The blue curve represents the median final (or cumulative) epidemic size (RH at the final time step). The green curve represents the median length of the epidemic (i.e., the point at which the number of infected individuals was below one). Each shaded area represents the 95% credible interval for the epidemiological indices ran under 50 different parameterizations of the life history traits. Here, simulations were run with the temperature conditions: Tmin = 10°C, Tmean = 25°C, and Tmax = 40°C (A) and Tmin = 20°C, Tmean = 25°C, and Tmax = 30°C (B). (TIF) [file pntd.0006451.s001.tif]

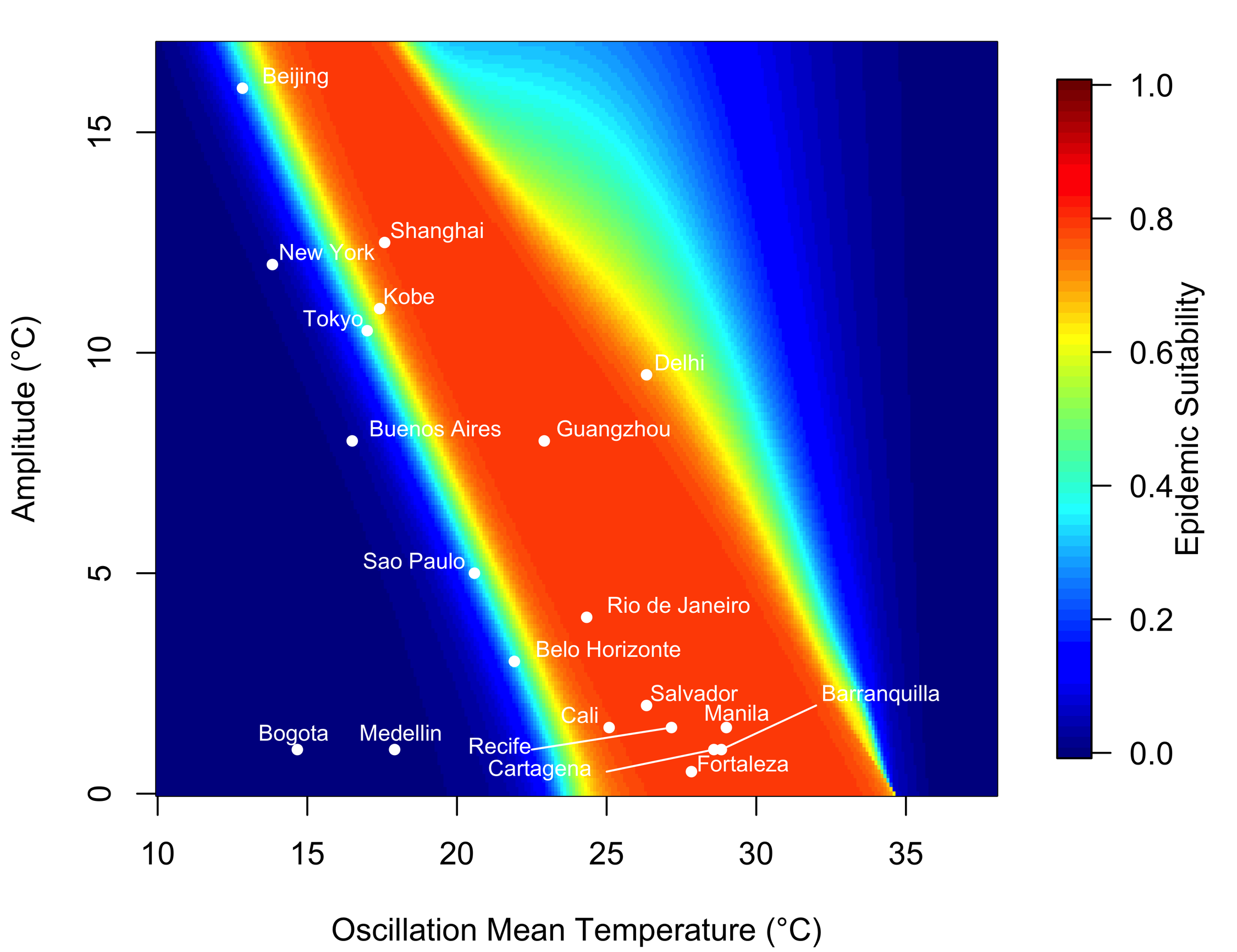

Supplement: S2 Fig — The heat map shows the epidemic suitability (represented as the proportion of the total human population infected during an epidemic) as a function of mean annual temperature and temperature range assuming 20% population immunity. Here, temperature range is defined as the seasonal variation about the annual mean temperature. Twenty large, globally important cities are plotted to illustrate their epidemic suitability. (TIF) [file pntd.0006451.s002.tif]

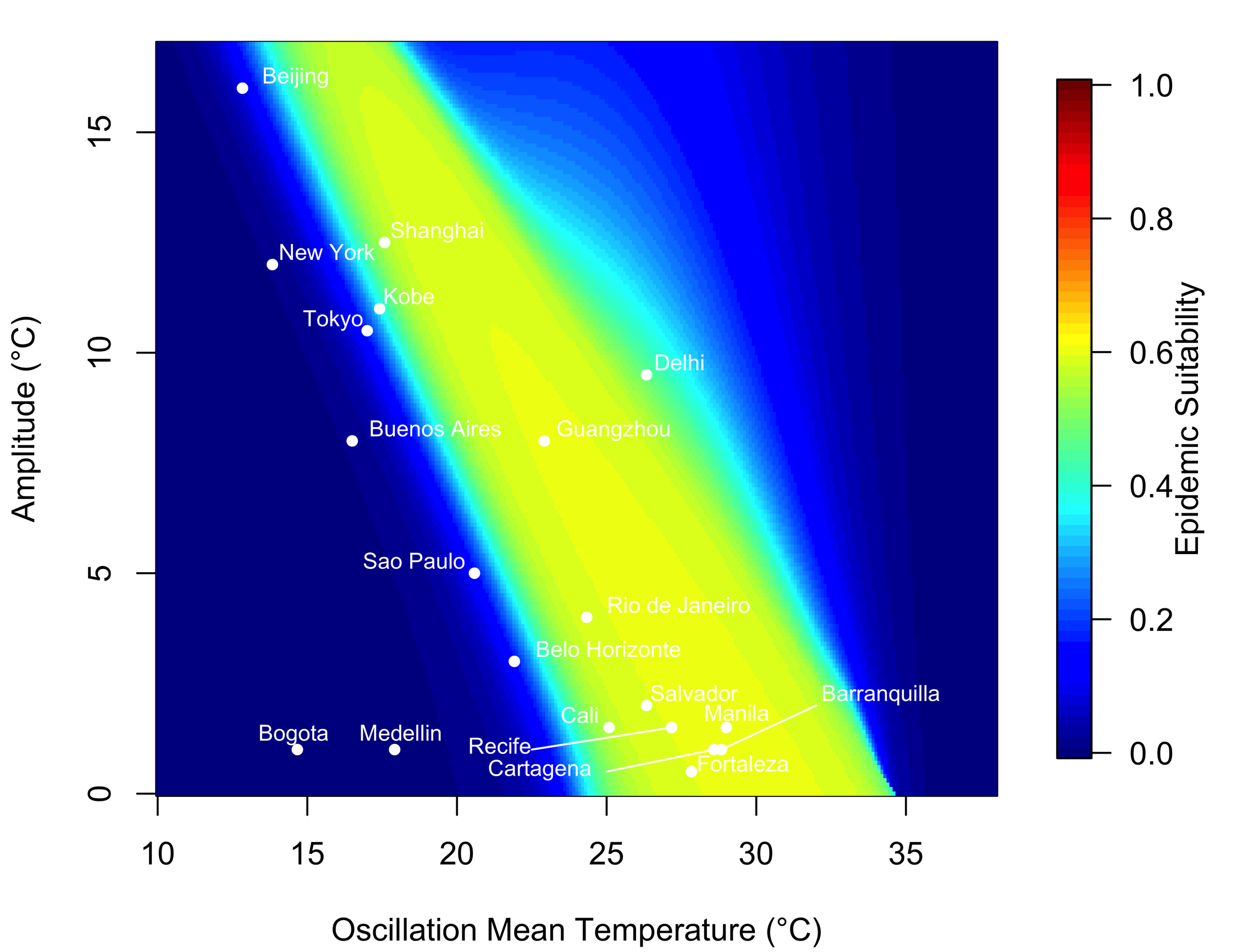

Supplement: S3 Fig — The heat map shows the epidemic suitability (represented as the proportion of the total human population infected during an epidemic) as a function of mean annual temperature and temperature range assuming 40% population immunity. Here, temperature range is defined as the seasonal variation about the annual mean temperature. Twenty large, globally important cities are plotted to illustrate their epidemic suitability. (TIF) [file pntd.0006451.s003.tif]

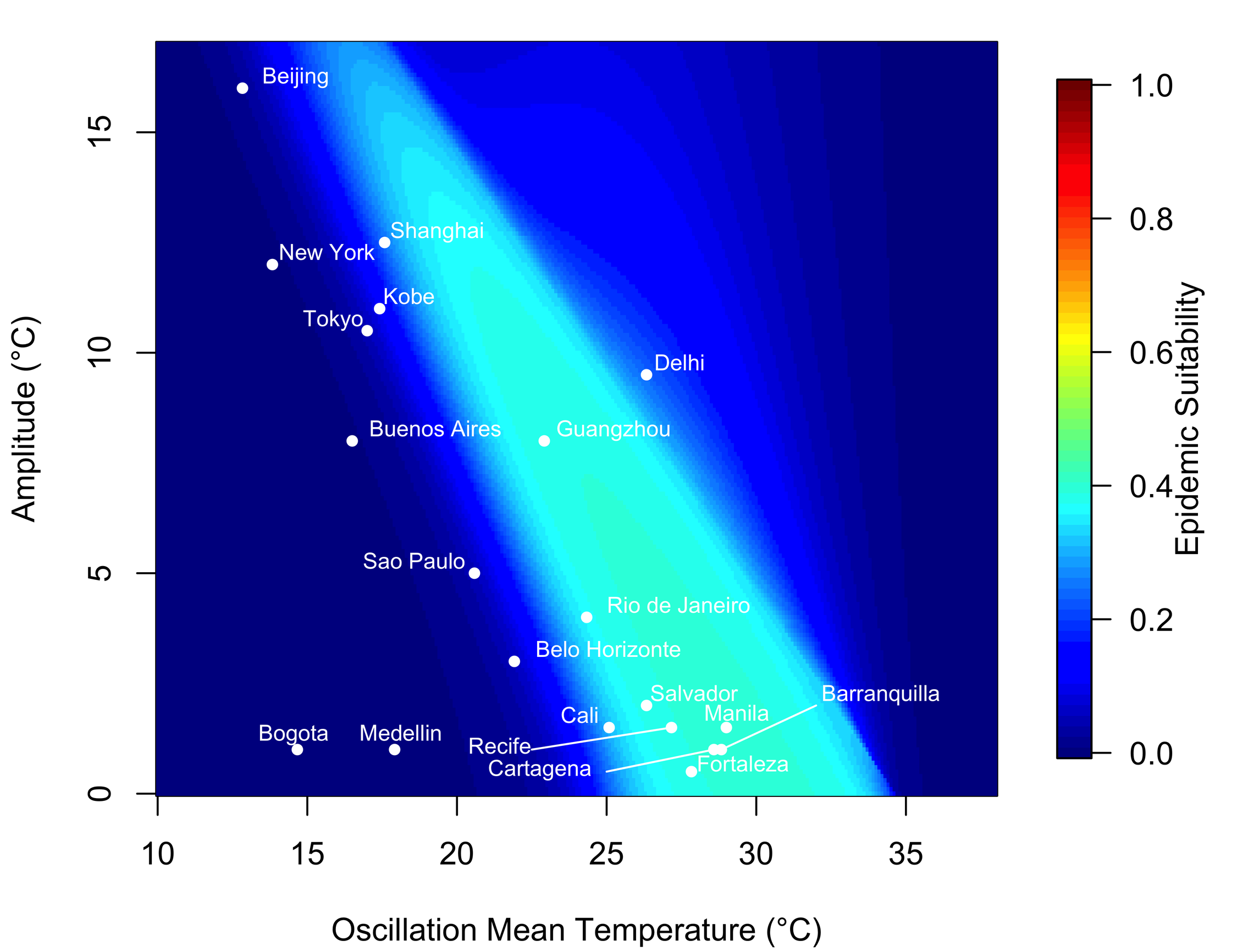

Supplement: S4 Fig — The heat map shows the epidemic suitability (represented as the proportion of the total human population infected during an epidemic) as a function of mean annual temperature and temperature range assuming 60% population immunity. Here, temperature range is defined as the seasonal variation about the annual mean temperature. Twenty large, globally important cities are plotted to illustrate their epidemic suitability. (TIF) [file pntd.0006451.s004.tif]

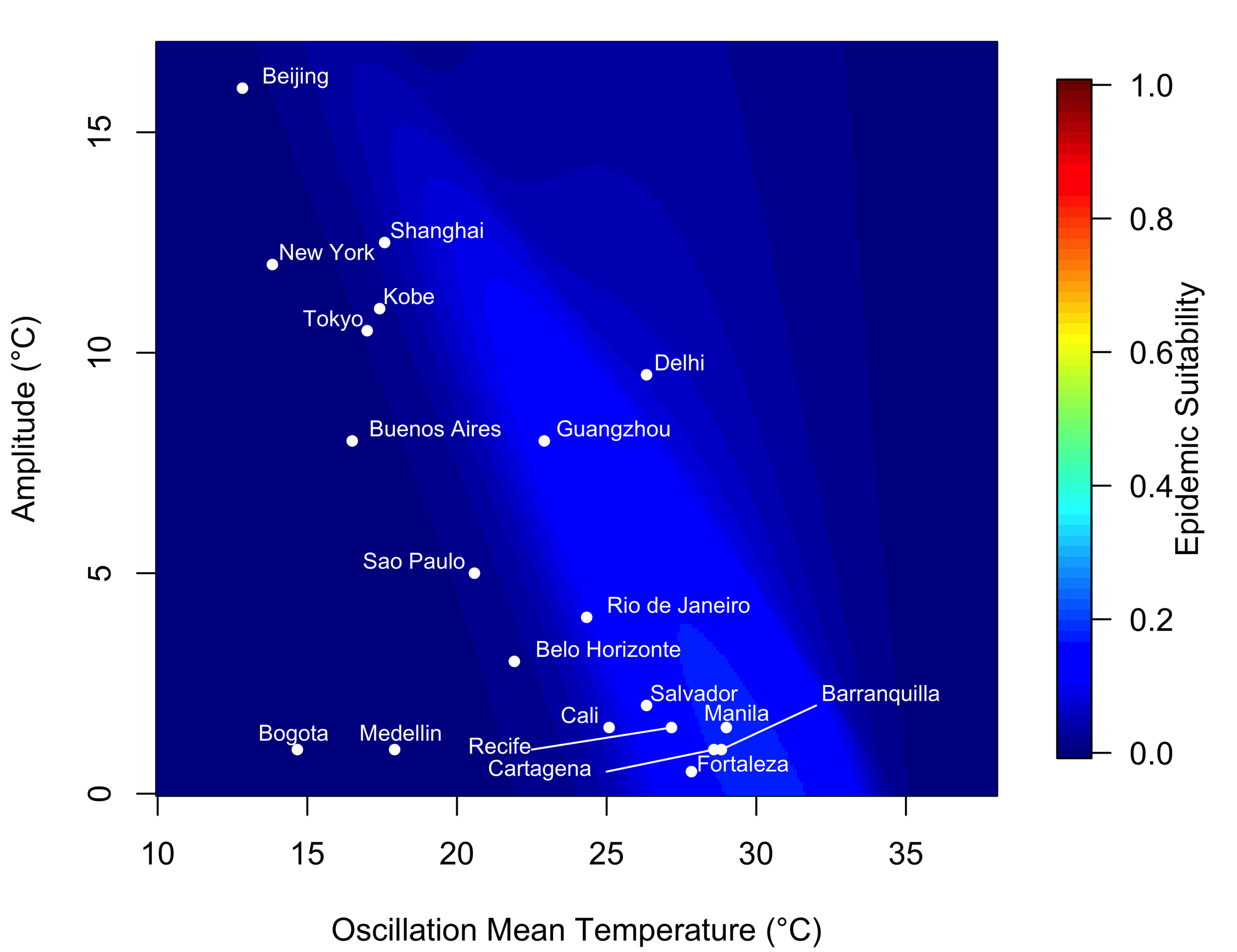

Supplement: S5 Fig — The heat map shows the epidemic suitability (represented as the proportion of the total human population infected during an epidemic) as a function of mean annual temperature and temperature range assuming 80% population immunity. Here, temperature range is defined as the seasonal variation about the annual mean temperature. Twenty large, globally important cities are plotted to illustrate their epidemic suitability. (TIF) [file pntd.0006451.s005.tif]

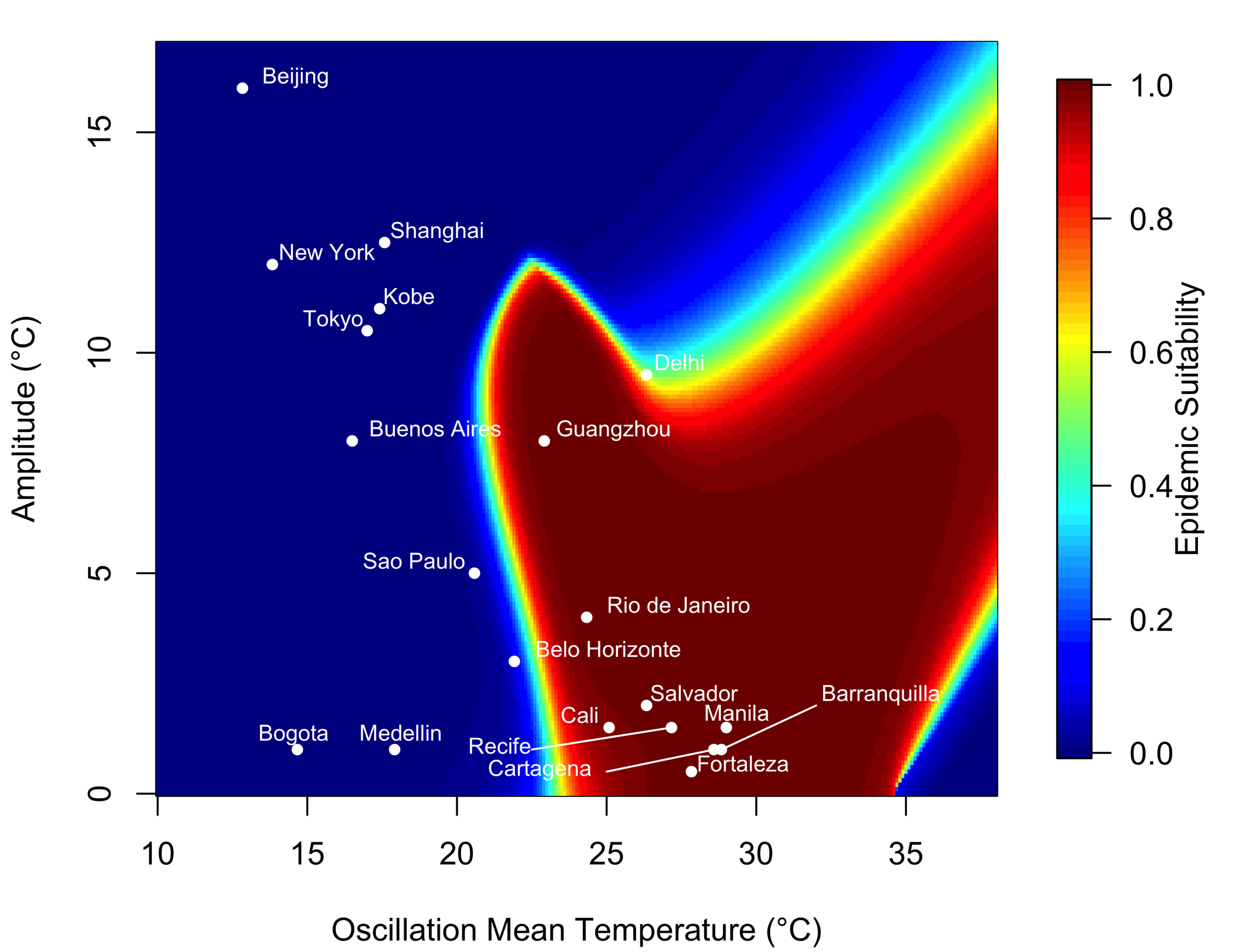

Supplement: S6 Fig — The heat map shows the epidemic suitability (represented as the proportion of the total human population infected during an epidemic) as a function of mean annual temperature and temperature range. Here, temperature range is defined as the seasonal variation about the annual mean temperature, and the simulation began at the minimum temperature of the regime. Twenty large, globally important cities are plotted to illustrate their epidemic suitability. (TIF) [file pntd.0006451.s006.tif]

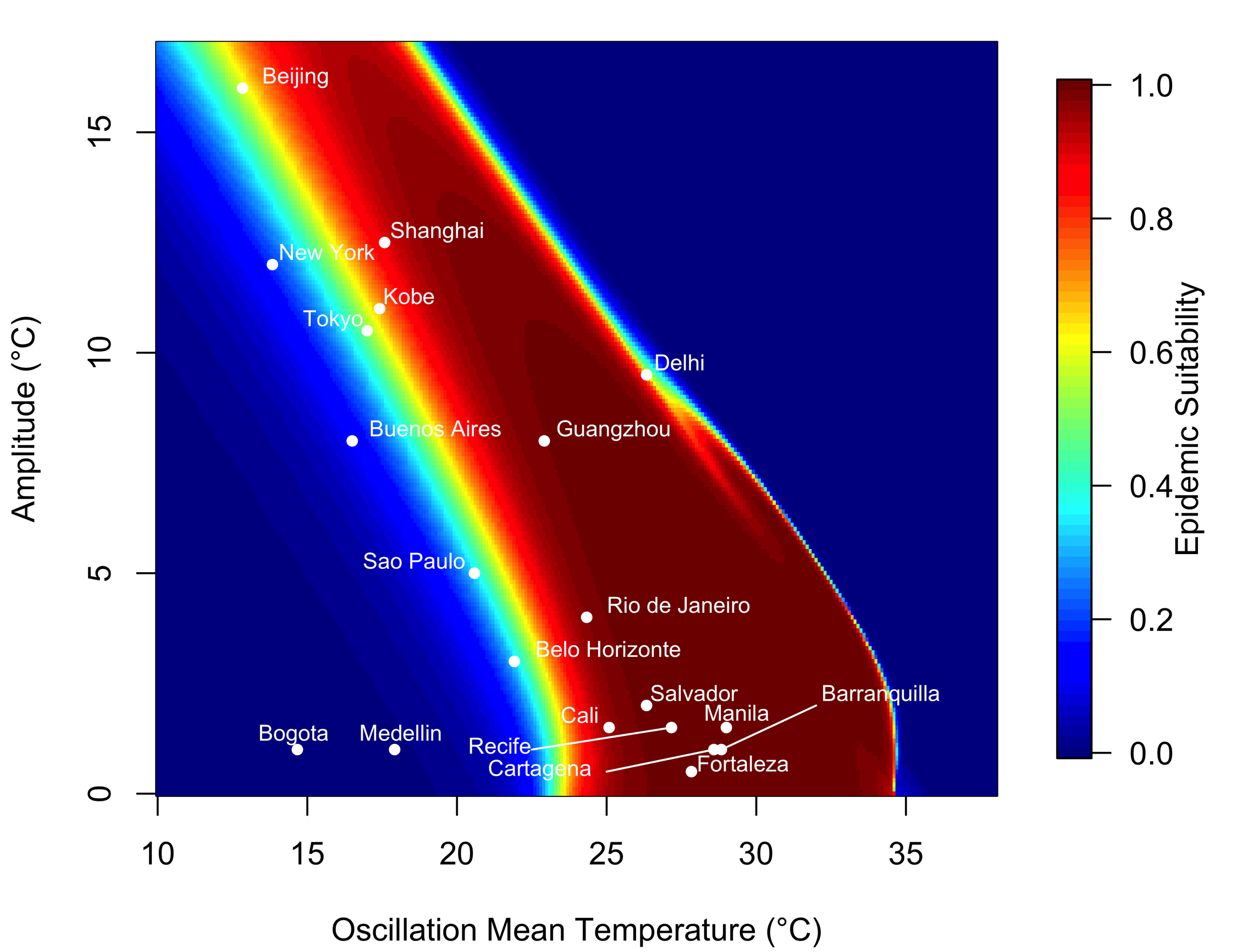

Supplement: S7 Fig — The heat map shows the epidemic suitability (represented as the proportion of the total human population infected during an epidemic) as a function of mean annual temperature and temperature range. Here, temperature range is defined as the seasonal variation about the annual mean temperature, and the simulation began at the maximum temperature of the regime. Twenty large, globally important cities are plotted to illustrate their epidemic suitability. (TIF) [file pntd.0006451.s007.tif]

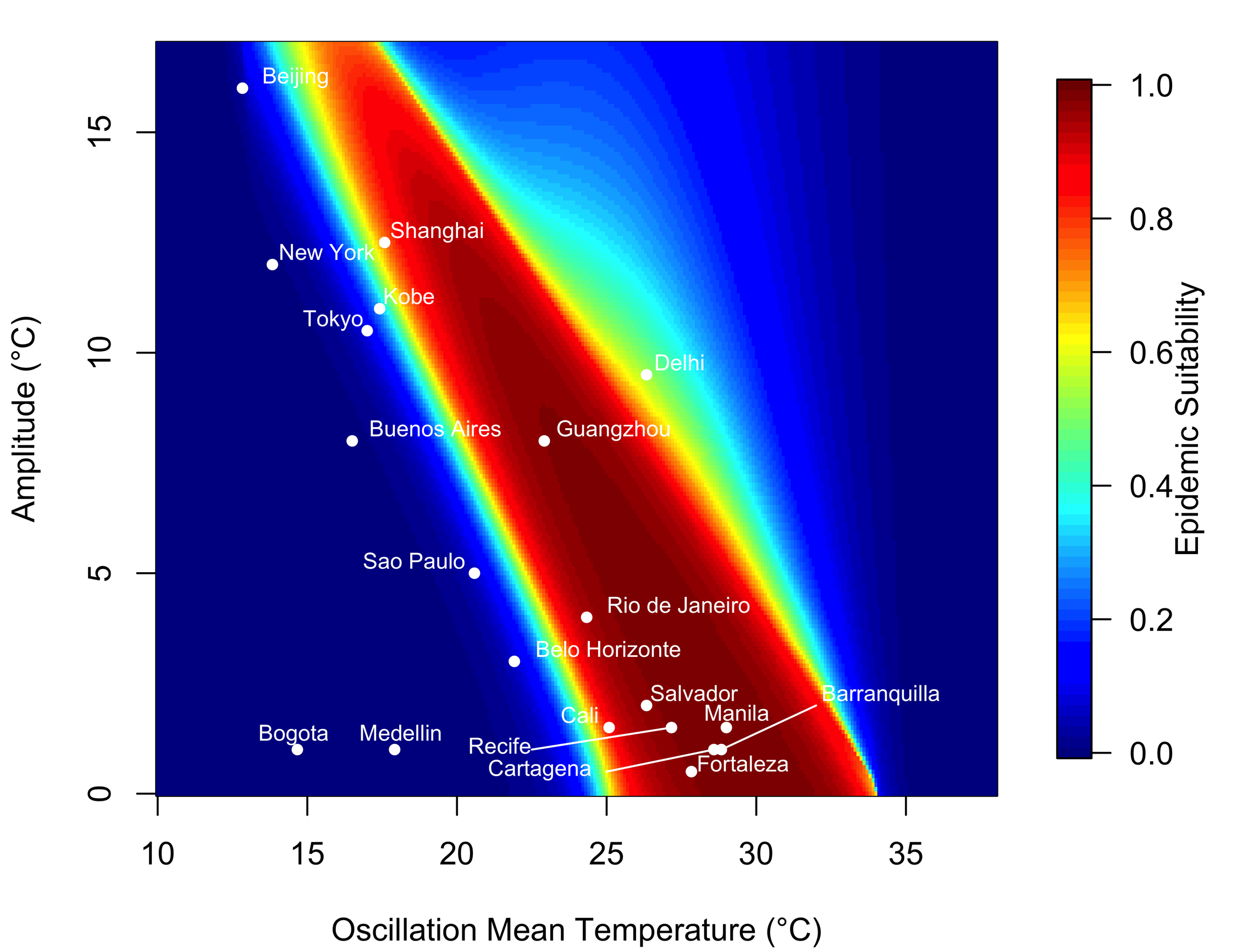

Supplement: S8 Fig — Epidemic suitability (represented as the proportion of the total human population infected during an epidemic) as a function of mean annual temperature and the temperature range. Temperature varied according to a seasonal temperature regime, and 50 samples of c, Tmin, and Tmax were taken from the joint posterior distribution of each trait thermal response from Mordecai et al. [24]. (TIF) [file pntd.0006451.s008.tif]

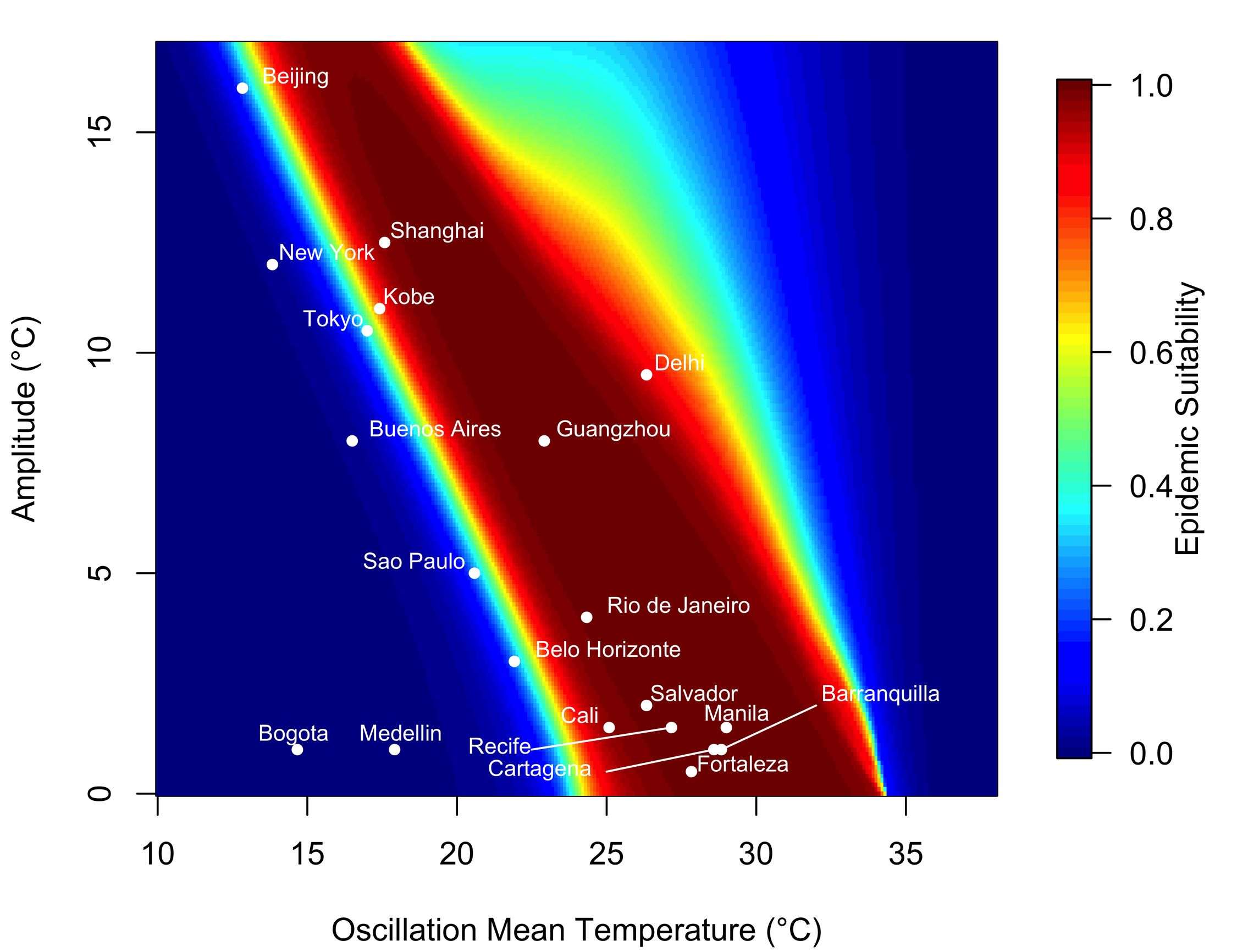

Supplement: S9 Fig — Epidemic suitability (represented as the proportion of the total human population infected during an epidemic) as mean annual temperature and the temperature range. Temperature varied according to a seasonal temperature regime, and 50 samples of c, Tmin, and Tmax were taken from the joint posterior distribution of each trait thermal response from Mordecai et al. [24]. (TIF) [file pntd.0006451.s009.tif]

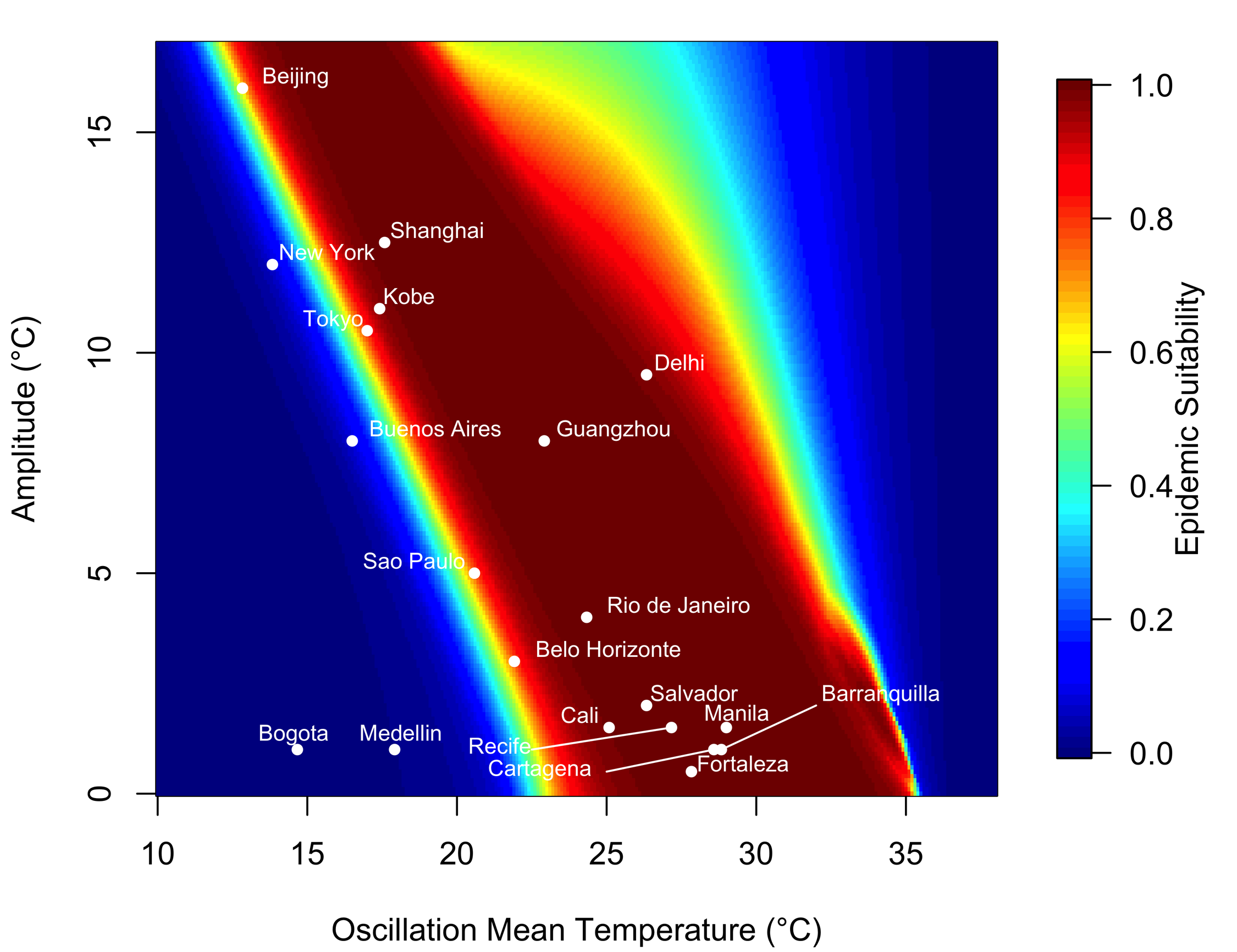

Supplement: S10 Fig — Epidemic suitability (represented as the proportion of the total human population infected during an epidemic) as mean annual temperature and the temperature range. Temperature varied according to a seasonal temperature regime, and 50 samples of c, Tmin, and Tmax were taken from the joint posterior distribution of each trait thermal response from Mordecai et al. [24]. (TIF) [file pntd.0006451.s010.tif]
