# Supplementary material for: Seasonal temperature variation influences climate suitability for dengue, chikungunya, and Zika transmission
Source: PLoS Negl Trop Dis. 2018 May 10;12(5):e0006451. doi: 10.1371/journal.pntd.0006451 (PMC5963813; doi:10.1371/journal.pntd.0006451)
Supplement: S1 Appendix — (PDF) [file pntd.0006451.s011.pdf]

Seasonal temperature variation influences climate suitability for dengue, chikungunya, and Zika transmission

John H. Huber, Marissa L. Childs, Jamie M. Caldwell, Erin A. Mordecai

Appendix

Functional Forms of Life History Traits

The functional forms of the life history traits used in this study were estimated by Mordecai et al [1]. Each life history trait was fit to either a quadratic or Brière function to reflect a symmetric or asymmetric nonlinear thermal response, respectively. The quadratic and Brière functions are parameterized by three values: a rate constant, a critical thermal minimum, and a critical thermal maximum.

The Brière function was introduced by Brière et al. [2] to characterize asymmetric, nonlinear thermal responses. This specific form was chosen to address four key requirements: (1) critical thermal minima and maxima, (2) an asymmetrical response at temperatures above and below the optimal temperature, (3) the presence of an inflection point, and (4) a marked decline in the life history trait as temperature exceeds the optimal temperature.

Starting Conditions

The model requires estimates of the mosquito-to-human ratio and the number of initially infectious humans and vectors to initiate each simulation. For these initial conditions, we used measures provided in previous models. In a model of the ZIKV outbreak in French Polynesia from 2013-2014, Kucharski et al. provided marginal posterior estimates for the

proportion of initially infectious mosquitoes and the number of initially infectious humans on each island [3]. We digitized the data for Tahiti and calculated the weighted average to arrive at estimates of 0.015 for the proportion of initially infectious vectors. The literature on the ratio of mosquitoes to humans is limited. One model [4] that measured mosquito oviposition behavior adopted a patch modeling framework in which the overall ratio of mosquitoes to humans was equal to two, which we adopted here.

With estimates for the mosquito-to-human ratio and the number of initially infectious individuals and vectors, we were able to calibrate our model. The total human population size, N_H, was set to a constant 10,000. Under the default assumption, we assumed that 1 individual was initially infectious, and the remaining population (9,999 individuals) was susceptible. E_H and R_H were initially set to zero. We then ran simulations assuming 20, 40, 60, and 80% of the population was immune at the simulation onset, respectively.

Since the carrying capacity and adult mosquito mortality rate were both dependent on temperature, the mosquito population size was subject to temperature effects. We assumed that the carrying capacity was greatest at 29°C (based on studies by Mordecai et al., [1] and Liu-Helmersson et al. [5]) and totaled approximately 20,000. To equilibrate the model, we set the initial mosquito population size (M_0) to the carrying capacity calculated from eq. 8 at the starting temperature. We assumed that 1.5% of mosquitoes were infected at the start of the epidemic, while the rest were susceptible [3]. The initial conditions were thus:

$$(S_V, E_V, I_V, S_H, E_H, I_H, R_H)_0 = (0.985M_0, 0, 0.015M_0, 9999, 0, 1, 0)$$

Simulations

Simulations were performed using a temperature-sensitive mechanistic modeling framework developed in C++. Code and supporting documentation for this model can be accessed at https://github.com/jhuber3/temperature-sensitive-sir. Simulation outputs were processed in the R Programming Language 4.3.1 [6].

References

1. Mordecai EA, Cohen JM, Evans M V., Gudapati P, Johnson LR, Lippi CA, et al. Detecting the impact of temperature on transmission of Zika, dengue, and chikungunya using mechanistic models. PLoS Negl Trop Dis. 2017;11. doi:10.1371/journal.pntd.0005568

2. Briere JF, Pracros P, Le Roux AY, Pierre JS. A novel rate model of temperature-dependent development for arthropods. Environ Entomol. 1999;28: 22–29. doi:10.1093/ee/28.1.22

3. Kucharski AJ, Funk S, Eggo RM, Mallet H-P, Edmunds WJ, Nilles EJ. Transmission Dynamics of Zika Virus in Island Populations: A Modelling Analysis of the 2013–14 French Polynesia Outbreak. Barker CM, editor. PLoS Negl Trop Dis. 2016;10: e0004726. doi:10.1371/journal.pntd.0004726

4. Le Menach A, McKenzie FE, Flahault A, Smith DL. The unexpected importance of mosquito oviposition behaviour for malaria: non-productive larval habitats can be sources for malaria transmission. Malar J. 2005;4: 23. doi:10.1186/1475-2875-4-23

5. Liu-Helmersson J, Stenlund H, Wilder-Smith A, Rocklöv J. Vectorial capacity of Aedes aegypti: Effects of temperature and implications for global dengue epidemic potential. PLoS ONE. 2014;9. doi:10.1371/journal.pone.0089783

6. Team RC. R: A Language and Environment for Statistical Computing. Vienna, Austria;

2017.
